# Comparison of high‐intensity sound and mechanical vibration for cleaning porous titanium cylinders fabricated using selective laser melting

**DOI:** 10.1002/jbm.b.33535

**Published:** 2015-10-01

**Authors:** Gary Seiffert, Carl Hopkins, Chris Sutcliffe

**Affiliations:** ^1^ Acoustics Research Unit, University of LiverpoolLiverpoolL69 3GHUK; ^2^ School of EngineeringUniversity of LiverpoolLiverpoolL69 3GHUK

**Keywords:** orthopedic components, acoustic cleaning, sonic cleaning, vibration, powder removal

## Abstract

Orthopedic components, such as the acetabular cup in total hip joint replacement, can be fabricated using porous metals, such as titanium, and a number of processes, such as selective laser melting. The issue of how to effectively remove loose powder from the pores (residual powder) of such components has not been addressed in the literature. In this work, we investigated the feasibility of two processes, acoustic cleaning using high‐intensity sound inside acoustic horns and mechanical vibration, to remove residual titanium powder from selective laser melting‐fabricated cylinders. With acoustic cleaning, the amount of residual powder removed was not influenced by either the fundamental frequency of the horn used (75 vs. 230 Hz) or, for a given horn, the number of soundings (between 1 and 20). With mechanical vibration, the amount of residual powder removed was not influenced by the application time (10 vs. 20 s). Acoustic cleaning was found to be more reliable and effective in removal of residual powder than cleaning with mechanical vibration. It is concluded that acoustic cleaning using high‐intensity sound has significant potential for use in the final preparation stages of porous metal orthopedic components. © 2015 Wiley Periodicals, Inc. J Biomed Mater Res Part B: Appl Biomater, 105B: 117–123, 2017.

## INTRODUCTION

There are significant advantages in fabricating orthopedic components from porous metals and alloys. The porosity and morphology are similar to those of cancellous bone; hence, the component has a low potential for stress shielding, and a high potential for both osseointegration and biological anchorage for tissue in‐growth, all of which result in the longevity of the implant when *in vivo*.^1^ Porous metals and alloys are manufactured using processes such as powder metallurgy,^2^ vacuum diffusion bonding of metal meshes,^3^ and selective laser melting (SLM).^4^, ^5^ These processes all leave residual powder within the component. For porous metal implants *in vivo*, any powder that leaves the implant and enters the periprosthetic tissue will cause an inflammatory response at the implantation site, which, in turn, may lead to osteoblastic bone resorption and fibrous tissue formation, resulting in loosening of the implant.^6^ Removal of residual powder from the tortuous paths within a porous metal component is, therefore, essential before it is implanted.

Standard industrial practice for powder removal in additive manufacturing involves: (1) brushing off loose powder, (2) blasting with inert gas, (3) bead blasting with various media, and (4) heat treatment to sinter on any remaining powder. In the case of metallic lattices, powder removal can be dealt with in a number of ways, including ultrasonication in water and by acid etching. To avoid wet processes, it would be advantageous to remove the majority of powder in the dry state. One possibility is to use high levels of mechanical vibration, but this could damage the component. This provides the motivation to assess a noncontact process using high‐intensity sound. Previous work^7^, ^8^ investigated the use of high‐intensity, low‐frequency sound to de‐bond powder layers from the collection plates of electrostatic precipitator air filters. In contrast to this previous application, orthopedic components can be placed inside the throat of a horn where there are extremely high sound pressure levels as well as turbulent airflow. This provides the novel aspect in this article where high‐intensity sound inside the horn has been investigated to determine the effect of sound pressure level, duration, and frequency content on the efficiency of powder removal from porous metals.

This work concerns the removal of residual powder from commercially‐pure titanium cylinders that were manufactured using SLM. The aims were: (a) to investigate the feasibility of acoustic cleaning using high‐intensity sound, (b) to investigate the influence of two acoustic cleaning process variables, (fundamental frequency of the horn and, for a given horn, the number of soundings) and (c) to compare high‐intensity sound and mechanical vibration.

## MATERIALS AND METHODS

This section describes the experimental procedures and equipment used to carry out the removal of powder from the test cylinders.

### Test specimens

**Figure 1:**
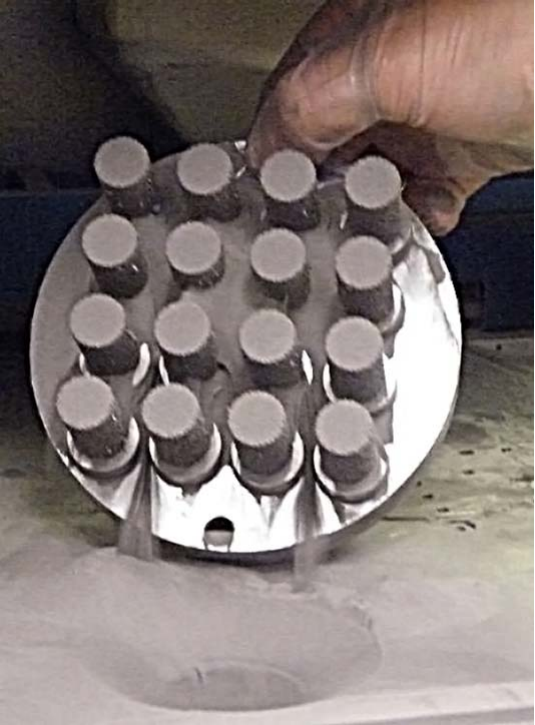
Sixteen test cylinders on a substrate holder showing surplus Ti powder pouring off at the end of the manufacturing process.

**Figure 2:**
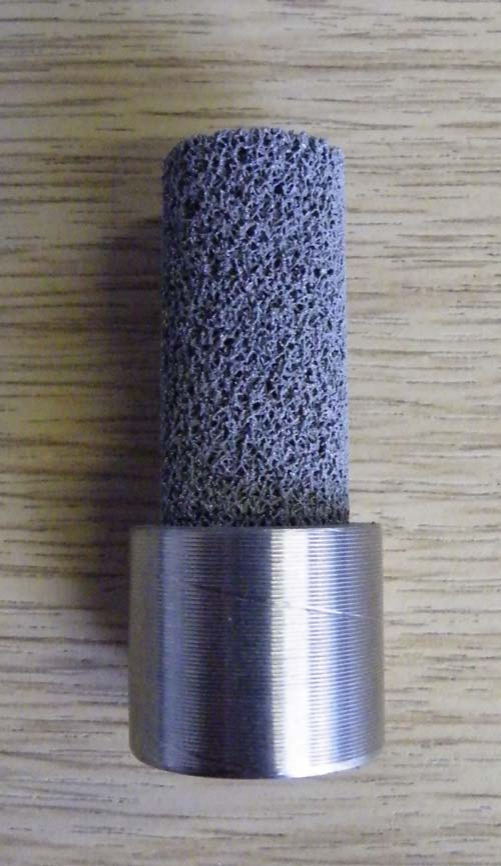
Close‐up of a single test cylinder on a minisubstrate.

Cylindrical test specimens (30 mm height and 15 mm outer diameter) were produced from commercially pure titanium (CpTi) built off a substrate (Figure 1) using an SLM100 machine (Realizer, Germany). The porous material was 30% randomized based on a tessellated octahedral matrix. Randomization was applied by shifting the nodal coordinate of a regular octahedral lattice by ±30% of the unit cell dimension.^9^ Each cylinder (Figure 2) had a porosity of ≈60% and a mean pore size of 450 μm. As the titanium powder had a tap density that is approximately 60% of the solid titanium, it was estimated that each cylinder could hold 5.7 g of powder.

### Air‐driven horns

Air‐driven horns are capable of generating high sound pressure levels, which cannot be created and/or sustained by standard loudspeakers. In this work, two industrial cleaning horns were used, the PAS 75 and PAS 230 (Primasonics International Ltd., Penrith, UK). The PAS 75 horn is made of fiber‐glass and is 3 m long with a 400 mm bell diameter and a fundamental frequency of 75 Hz.

The PAS 230 is a spun aluminium horn, 0.62 m long with a 208 mm bell diameter and a fundamental frequency of 230 Hz. These were considered because (a) horns with low fundamental frequencies are usually long, and this might not be practical to implement in a manufacturing process and (b) it is not known whether the efficacy of acoustic cleaning depends on frequency. Each of the horns was driven by compressed air, delivered from a compressor (air pressure = 1.03 MPa) for which the air reservoir gave a 12 s sounding.

### Equipment for measurement of high sound pressure levels inside horns

Conventional measurement microphones with thin metal diaphragms cannot be used to measure the sound pressure level either inside or near the mouth of the horn because the high‐intensity sound and turbulent nature of the field would destroy the diaphragm. Hence, a piezoelectric hydrophone (Type 8103 Brüel & Kjær, Nærum, Denmark) was connected to a signal conditioning amplifier (Nexus Type 2692 Brüel & Kjær) to measure the narrow band spectra and maximum sound pressure levels. Hydrophones are designed to be used in water; hence, the sensitivity in air was established by comparison against calibrated measurement microphones in the reverberation chamber (volume 122 m^3^) with a broadband white noise source, power amplifier, and loudspeaker (100 Hz to 10 kHz). The hydrophone was found to be accurate to within 1 dB, which was sufficient to enable precise and repeatable measurements in air.

### Measurement of sound pressure levels inside horns

**Figure 3:**
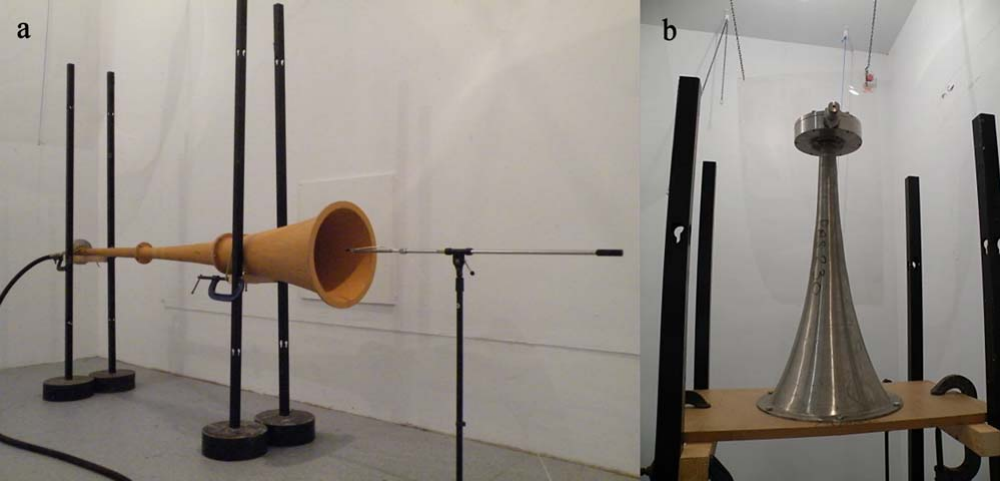
Horns installed in the reverberation chamber. (a) The PAS 75 horn with the hydrophone fixed to a boom, allowing sampling at different positions inside the horn. (b) The PAS 230 horn supported by a wooden plate (Note: a circular aperture is underneath the horn to allow normal operation).

To reduce sound transmission to areas outside the laboratory, the horns were installed in a reverberation chamber. The PAS 75 horn was mounted horizontally with the hydrophone located at the mouth of the horn [Figure 3(a)]. The hydrophone was moved 100 mm inside the horn, and the measurement repeated. This process was repeated using 100 mm increments inside the horn until it was 1.5 m from the mouth of the horn. The PAS 230 horn was mounted vertically [Figure 3(b)], and the hydrophone was moved in 100 mm intervals until it was 500 mm from the mouth of the horn. Beyond 500 mm, the horn diameter reduces to <60 mm, which was too small a space to position and fix the test cylinders. The horn was sounded for 12 s, but the equivalent continuous sound pressure level, *L*_eq,10s_, was measured over a 10‐s time period to avoid the initial rise and decay of the sounding.

### Acoustic cleaning

**Figure 4:**
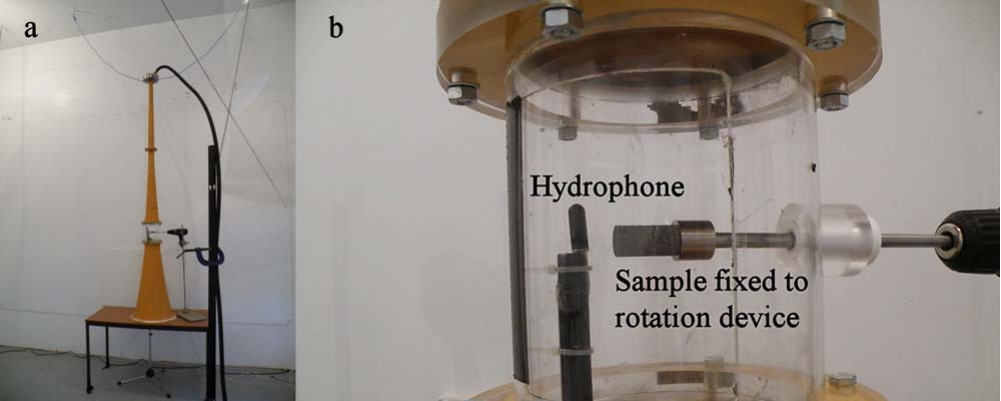
(a) Modified PAS 75 horn with the cleaning chamber fitted. Experimental set‐up. (b) A test cylinder installed in the cleaning chamber close to the hydrophone.

A cleaning chamber was designed and constructed that fitted into the midsection of the PAS 75 horn [Figure 4(a,b)]. This modified PAS 75 allowed the sample to be exposed to higher sound pressure levels inside the horn than occurred at the mouth. There are practical limitations on the position of the cleaning chamber along the horn. The chosen position allowed insertion of a chamber before the throat narrowed toward the driver; the distance from the midpoint of the chamber to the mouth of the horn being 870 mm. This chamber was transparent to enable visual inspection of the porous metal sample. While the horn was sounding, the sample was rotated in the sound field to facilitate maximum removal of powder by exposing all sides of the sample to the incident sound and allowing the powder to exit from all surfaces of the cylinder. The horn was mounted above a circular aperture in order not to impede air flow [Figure 4(a)]. This allowed the dislodged powder from the cylinder to fall under gravity away from the sample.

The PAS 230 horn was smaller than the PAS 75; hence, it was not practical to install a cleaning chamber. Therefore, the cylinder was positioned at the mouth of the PAS 230. As with the PAS 75, the PAS 230 was mounted above a circular aperture [Figure 3(b)].

Three test cylinders were weighed using a precision balance (precision: ±0.01 g) and positioned inside the cleaning chamber. Each cylinder was rotated at 12 rpm, and the horn was sounded for 12 s after which the cylinder was removed from the chamber and weighed. This process was repeated after two, three, four, and five soundings. For one of the cylinders, the horn was sounded 20 times to check whether there was any advantage in increasing the exposure. Furthermore, one cylinder was installed in the cleaning chamber and rotated for 12 s without the horn sounding.

### Micro‐computed tomography examination of acoustic cleaned cylinder

Before and after acoustic cleaning using the PAS 75 horn, the morphology of one test cylinder was examined using micro‐computed tomography (CT).^10^ The samples were scanned at a resolution of 9 μm per voxel using a commercial micro‐CT unit (Phoenix v|tome|x, GE Measurement and Control, Boston, MA). The X‐ray tube voltage and filament current were fixed at 100 kV and 70 μA, respectively. A rotation step of 0.5° was set within an angular range of 360°.

### Cleaning using mechanical vibration

In a previous work,^7^ sinusoidal signals were used to identify the vibration level required to de‐bond powdered material that had been electrostatically deposited onto a metal surface. This required acceleration levels between 153 and 164 dB re 10^−6^ m/s^2^, which corresponded to peak acceleration values in the range of 63–224 m/s^2^. To remove metal powders from SLM‐fabricated specimens, it was assumed that the required acceleration levels would be higher than the aforementioned values and that mechanical rapping (rather than sinusoidal excitation) would be beneficial in shaking/removing loose powder from the voids within the porous specimen.

**Figure 5:**
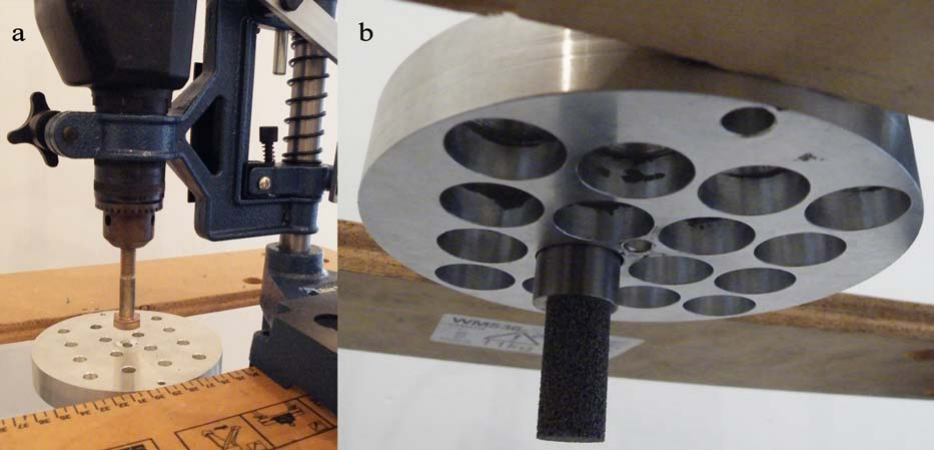
Mechanical vibration. (a) Hammer drill used to apply vibration to the upper surface of the test cylinder holder. (b) A single test cylinder fixed to the underside of the sample holder.

A hammer drill was used to vibrate the underside of the test cylinder holder [Figure 5(a)]. Into this holder, an inverted sample was fixed by a screw in its substrate. As vibration was applied, the powder fell under gravity into a collection beaker. During the process, an accelerometer (Type 4374 Brüel & Kjær), which was fixed to the base of each sample, measured an overall acceleration level of 170 dB re 10^−6^ m/s^2^ corresponding to a peak acceleration of 449 m/s^2^. Three test cylinders were cleaned, with each being vibrated for 10 s in the first instance and, then, for a further 10 s.

### Statistical analysis

Analysis for statistical significance was carried out using the Kruskal–Wallis test, and differences were considered statistically significant when the *p*‐values were less than 0.05.

## RESULTS

**Figure 6:**
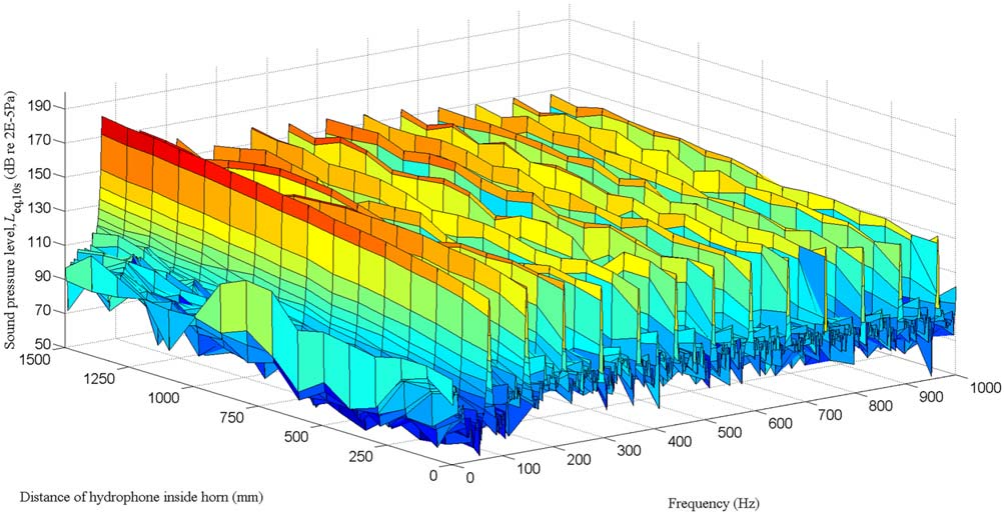
Sound pressure level measured at distances up to 1500 mm inside the PAS 75 horn.

**Figure 7:**
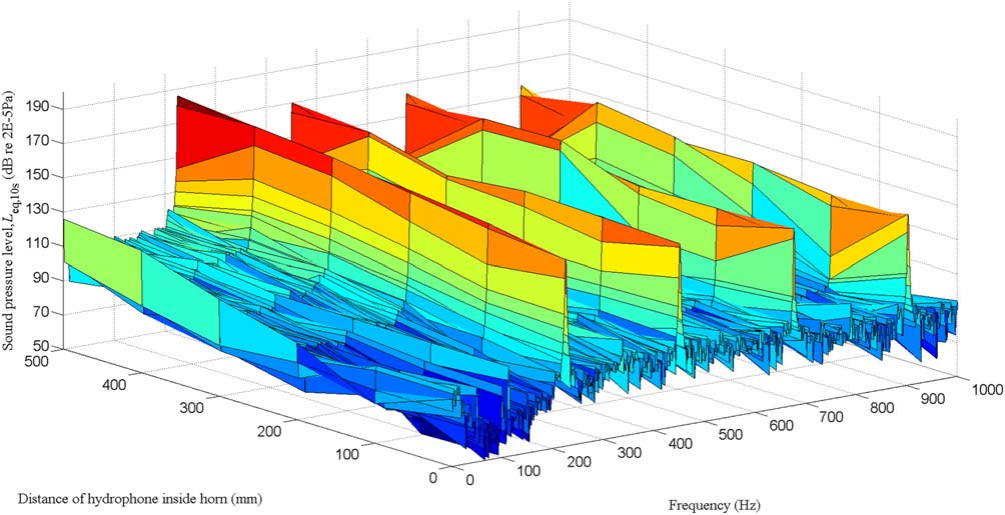
Sound pressure level measured at distances up to 500 mm inside the PAS 230 horn.

**Figure 8:**
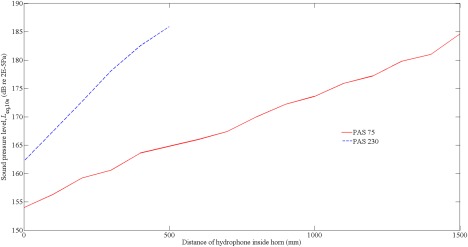
Variation of maximum sound pressure level with distance inside the PAS 75 and PAS 230 horns, measured from the mouth of the horn.

For the PAS 75, the first peak in the narrow band spectrum occurred at the fundamental frequency (75 Hz), with subsequent peaks occurring at the harmonics (Figure 6). The overall sound pressure level 1500 mm inside the horn was dominated by the level at the fundamental frequency because the harmonics were at least 10 dB lower. At the mouth of the horn (0 mm), the first two harmonics are at a higher level than the fundamental. In contrast, the harmonics for the PAS 230 all had lower levels than the fundamental (Figure 7). Hence, it is more instructive to refer to the overall sound pressure level rather than quote levels at individual frequencies. For both horns, the overall level increased with increasing distance inside the horn (Figure 8). The PAS 230 had higher overall levels than the PAS 75; this was attributed to the rigidity of the PAS 230, which was made of Al rather than the PAS 75, which was fiber glass.

**Figure 9:**
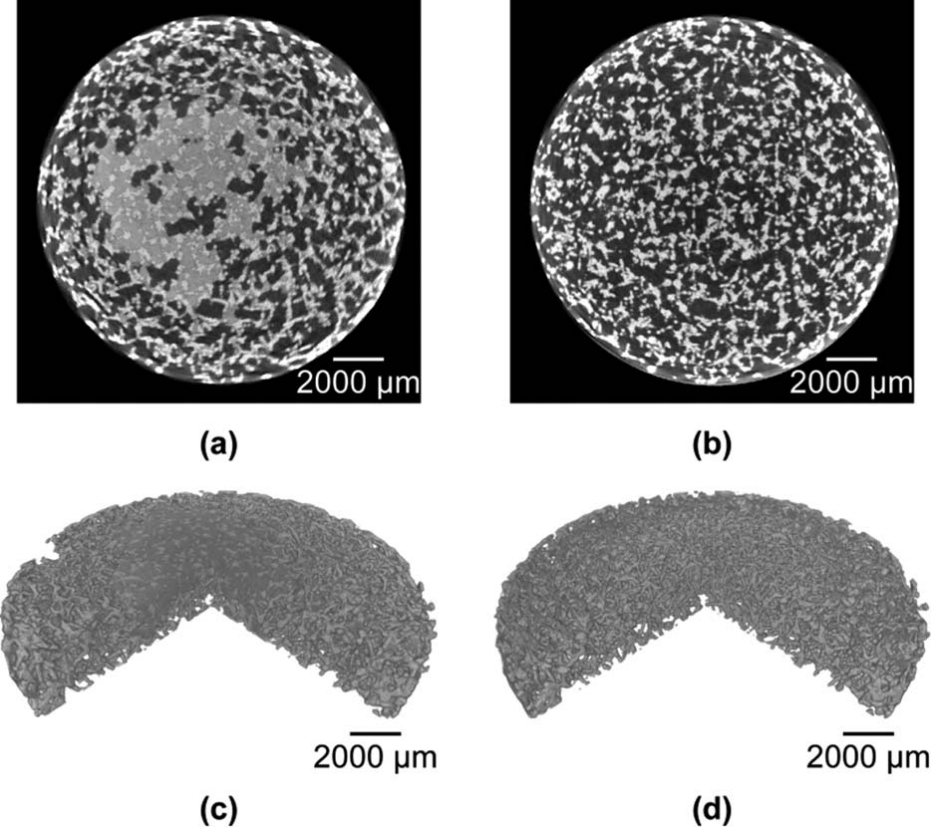
Micro‐CT scans of one of the test cylinders. (a, c) Before acoustic cleaning. (b, d) After acoustic cleaning in the cleaning chamber of the PAS 75 horn.

Acoustic cleaning with the PAS 75 and PAS 230 (overall sound pressure levels of 170 and 165 dB, respectively) removed 5.8 g and 5.9 g (average values), respectively, of loose powder from the cylinders after five soundings (Table 1). This corresponded to the estimated weight of residual powder (5.7 g). After four soundings, there was no measurable change in the mass of powder removed (Table 1). For one of the cylinders, this was checked with 20 soundings, and there was no change in the specimen mass. The mass of powder removed did not significantly differ between the PAS 75 and PAS 230 horns (*H*(1) = 0.048, *p* = 1), even though the horns had different fundamental frequencies. The micro‐CT scans (Figure 9) provide visual confirmation that acoustic cleaning removes the powder near the centre of the cylinder.

**Table 1:** Summary of Test Results from Acoustic Cleaning After Exposure to 170 dB Inside the PAS 75 Horn

Specimen	Initial Mass (g)	Mass After Sounding No.1	Mass After Sounding No.2	Mass After Sounding No.3	Mass After Sounding No.4	Mass After Sounding No.5	Mass of Powder Removed After Five Soundings (g)
A	38.62	32.81	32.78	32.76	32.75	32.75	5.87
B	37.39	31.94	31.93	31.92	31.91	31.91	5.48
C	38.29	32.32	32.28	32.27	32.27	32.27	6.02

Note that the mass corresponds to the cylinder and substrate.

The process of installing a cylinder in the cleaning chamber and rotating it for 12 s without the horn sounding resulted in a weight reduction of 0.17 g, which was negligible compared with the weight of powder removed by acoustic cleaning.

Compared with acoustic cleaning with the two horns, 20 s of mechanical vibration is less reliable and less effective (Tables 1, 2, 3), and the two approaches are significantly different, *H*(1) = 5.4, *p* = 0.024. Application times of 10 and 20 s for mechanical vibration had negligible effect on the amount of residual powder removed (Table 3).

**Table 2:** Summary of test results from acoustic cleaning after exposure to 165 dB at the mouth of the PAS 230 horn

Specimen	Initial Mass (g)	Mass After Sounding No.1	Mass After Sounding No.2	Mass After Sounding No.3	Mass After Sounding No.4	Mass After Sounding No.5	Mass of Powder Removed After Five Soundings (g)
D	38.53	32.66	32.4	32.38	32.38	32.38	6.15
E	38.01	32.2	32.19	32.18	32.16	32.16	5.85
F	38.44	32.76	32.76	32.74	32.73	32.73	5.71

Note that the mass corresponds to the cylinder and substrate.

**Table 3:** Summary of Test Results from Cleaning Using Mechanical Vibration

Specimen	Initial Mass (g)	Mass After Vibration for 10 s (g)	Mass After Vibration for 20 s (g)	Mass of Powder Removed After Vibration for 20 s (g)
G	36.04	32.20	32.18	3.86
H	36.95	32.82	32.79	4.16
I	37.97	32.87	32.86	5.11

Note that the mass corresponds to the cylinder and substrate.

## DISCUSSION

Acoustic cleaning with both horns removed the predicted mass of loose powder, which indicates that the influence of the fundamental frequency is not significant. Previous work^1^ on electrostatically deposited fly‐ash, calcium sulfate, and calcium carbonate on a flat, metal surface showed that mean de‐bonding sound pressure levels were all below 151 dB between 75 and 300 Hz. This work used much higher overall sound pressure levels of 165 and 170 dB. Hence, although the acoustic forces are frequency‐dependent, the overall force due to the overall sound pressure level was able to overcome the adhesive forces between Ti particles and the matrix (and possibly the cohesive forces between Ti particles as well). Theoretical prediction^1^ of the acoustic forces needed to remove powder particles from large plane surfaces requires knowledge of the Hamaker constant. Unfortunately, this constant is not known for Ti, and any prediction based on large plane surfaces is highly unlikely to be relevant to porous metals.

To the authors’ knowledge, this is the first study to investigate acoustic cleaning of porous objects inside acoustic horns. One of the limitations in this study was the small number of specimens that were tested using a specific porous matrix. However, for all the cylinders that were acoustically cleaned, the majority of loose powder was removed after one 12 s sounding, and after four soundings, there was no measurable change in the mass of powder. Therefore 48 s of acoustic cleaning is sufficient.

## CONCLUSIONS

Acoustic cleaning using high‐intensity sound inside horns was shown to remove all of the residual, loose powder inside porous Ti cylinders that were fabricated using SLM. The efficiency of this process is not influenced by the fundamental frequency of the horn used (75 vs. 230 Hz) or, for a given horn, the number of soundings (between 1 and 20). Furthermore, for these cylinders, acoustic cleaning was more reliable and effective than cleaning using mechanical vibration and avoids the risk of damaging the porous metal. These findings suggest that acoustic cleaning has significant potential for the final preparation of porous metal orthopedic components, such as acetabular cups in total hip joint replacements, and tibial trays in total knee joint replacements, before implantation in the body.

## References

[1] Lewis G. Properties of open‐cell porous metals and alloys for orthopaedic applications. J Mater Sci Mater Med 2013;24:2293–2325. 23851927 10.1007/s10856-013-4998-y

[2] Pilliar RM. Powder metal‐made orthopedic implants with porous surface for fixation by tissue ingrowth. Clin Orthop Relat Res 1983;176:42–51. 6851341

[3] Martell JM , Pierson R , Jacobs JJ , Rosenberg AG , Maley M , Galante JO. Primary total hip reconstruction with a titanium fiber‐coated prosthesis inserted without cement. J Bone Joint Surg Am 1993;75(4):554–571. 8478383 10.2106/00004623-199304000-00010

[4] Stamp R , Fox P , O'Neill W , Jones E , Sutcliffe C. The development of a scanning strategy for the manufacture of porous biomaterials by selective laser melting. J Mater Sci Mater Med 2009;20:1839–1848. 19536640 10.1007/s10856-009-3763-8

[5] Mour M , Das D , Winkler T , Hoenig E , Mielke G , Morlock M , Schilling A. Advances in porous biomaterials for dental and orthopaedic applications. Materials 2010;3:2947–2974.

[6] Bi Y , Van De Motter RR , Ragab AA , Goldberg VM , Anderson JM , Greenfield EM. Titanium particles stimulate bone resorption by inducing differentiation of murine osteoclasts. J Bone Joint Surg Am 2001;83:501–501. 11315778 10.2106/00004623-200104000-00004

[7] Seiffert G , Gibbs BM. Removal of electrostatically deposited powders using high intensity low frequency sound. Part 1: Experimental deposition and removal. J Low Freq Noise Vib Act Control 2010;29:171–187.

[8] Seiffert G , Gibbs BM. Removal of electrostatically deposited powders using high intensity low frequency sound. Part 2: Quantification of adhesive and cohesive forces using vibration. J Low Freq Noise Vib Act Control 2010;29:267–279.

[9] Mullen L , Stamp RC , Fox P , Jones E , Ngo C , Sutcliffe CJ. Selective laser melting: A unit cell approach for the manufacture of porous, titanium, bone in‐growth constructs, suitable for orthopedic applications. II. Randomized structures. J Biomed Mater Res Part B: Appl Biomater 2010;92:178–188. 19810111 10.1002/jbm.b.31504

[10] Zhang Z , Jones D , Yue S , Lee PD , Jones JR , Sutcliffe CJ , Jones E. Hierarchical tailoring of strut architecture to control permeability of additive manufactured titanium implants. Mater Sci Eng C 2013;33 (7):4055–4062. 10.1016/j.msec.2013.05.050 23910314

